# Complete gene expression profiling of *Saccharopolyspora erythraea *using GeneChip DNA microarrays

**DOI:** 10.1186/1475-2859-6-37

**Published:** 2007-11-26

**Authors:** Clelia Peano, Silvio Bicciato, Giorgio Corti, Francesco Ferrari, Ermanno Rizzi, Raoul JP Bonnal, Roberta Bordoni, Alberto Albertini, Luigi Rossi Bernardi, Stefano Donadio, Gianluca De Bellis

**Affiliations:** 1 Institute for Biomedical Technologies, National Research Council, Milan, Italy; 2 Department of Chemical Engineering Processes, University of Padova, Padova, Italy; 3 Department of Biomedical Sciences, University of Modena and Reggio Emilia, Modena, Italy; 4 University of Milan, Department of Biomedical Sciences and Technologies, Milan, Italy; 5 KtedoGen, Milan, Italy

## Abstract

**Background:**

The *Saccharopolyspora erythraea *genome sequence, recently published, presents considerable divergence from those of streptomycetes in gene organization and function, confirming the remarkable potential of *S. erythraea *for producing many other secondary metabolites in addition to erythromycin. In order to investigate, at whole transcriptome level, how *S. erythraea *genes are modulated, a DNA microarray was specifically designed and constructed on the *S. erythraea *strain NRRL 2338 genome sequence, and the expression profiles of 6494 ORFs were monitored during growth in complex liquid medium.

**Results:**

The transcriptional analysis identified a set of 404 genes, whose transcriptional signals vary during growth and characterize three distinct phases: a rapid growth until 32 h (Phase A); a growth slowdown until 52 h (Phase B); and another rapid growth phase from 56 h to 72 h (Phase C) before the cells enter the stationary phase. A non-parametric statistical method, that identifies chromosomal regions with transcriptional imbalances, determined regional organization of transcription along the chromosome, highlighting differences between core and non-core regions, and strand specific patterns of expression. Microarray data were used to characterize the temporal behaviour of major functional classes and of all the gene clusters for secondary metabolism. The results confirmed that the *ery *cluster is up-regulated during Phase A and identified six additional clusters (for terpenes and non-ribosomal peptides) that are clearly regulated in later phases.

**Conclusion:**

The use of a *S. erythraea *DNA microarray improved specificity and sensitivity of gene expression analysis, allowing a global and at the same time detailed picture of how *S. erythraea *genes are modulated. This work underlines the importance of using DNA microarrays, coupled with an exhaustive statistical and bioinformatic analysis of the results, to understand the transcriptional organization of the chromosomes of micro-organisms producing natural products.

## Background

Soil-inhabiting *Actinomycetes *are prominent antibiotic producers. Berdy [1] estimated that 8700 antibiotics have been discovered from them, compared with 2900 from all other bacteria and 4900 from fungi. Even including secondary metabolites with biologic activities other than anti-microbial, *Actinomycetes *still stand out as excellent producers, with *Streptomyces *being the most prolific genus. *Actinomycetes *are an abundant and diverse group, encompassing several different genera belonging to diversified families within the order *Actinomycetales *[2]. The large number of secondary metabolites produced by these bacteria probably correlates to the competitive environment where they strive.

The empirical observation that *Actinomycetes *are excellent secondary metabolite producers has been confirmed by the first genomic studies, which have revealed that these filamentous bacteria have the genetic potential to produce tens of different metabolites [3-5], in contrast with most other bacterial phyla [6]. In addition, this seems to be a taxon-related feature, since also the obligate marine actinomycete *Salinispora arenicola *harbors the potential to make many different secondary metabolites [7]. It has been suggested that the ability to deploy a differentiated chemical arsenal may be the general evolutionary strategy employed by many representatives of the *Actinomycetales *that grow as filamentous mycelia in highly competitive environments [8].

The model actinomycete *Streptomyces coelicolor *has been subjected to intensive studies for over 40 years [9]. The availability of the 8.7-Mb *S. coelicolor *chromosomal DNA sequence [3] and the development of efficient methods for genome-wide analysis of expression profiles using DNA microarrays [10] enabled to simultaneously and globally assess factors that affected transcription of *Streptomyces *genes and regulatory pathways. A global analysis of growth phase gene expression and of the regulation of the biosynthetic pathways has been performed [11] and a regional organization of gene expression profiling inferred [12].

However, while many producer strains have been subjected to physiological studies and to strain improvement programs for optimizing production, little has been reported on this work. Consequently, there is fragmentary information on antibiotic production in *Actinomycetes *and we still do not know how applicable the *S. coelicolor *model is to distantly related industrial strains. For example, it is not known if lack of success, in industrial strain improvement programs, have resulted in the lack of titre increases obtained by the manipulation of pathway specific or global regulatory genes, which have otherwise been successful in academia [13,14].

Recently, Oliynyk et al. [15] reported the genome sequence of *Saccharopolyspora erythraea *NRRL2338, a mycelium-forming actinomycete and the producer of the clinically important macrolide antibiotic erythromycin A. *S. erythraea*, albeit originally identified as *Streptomyces erythreus*, is only distantly related to *S. coelicolor*, since *Saccharopolyspora *and *Streptomyces *are two distinct genera belonging to the suborders *Pseudonocardinae *and *Streptomycinae*, respectively. Consequently, the *S. erythraea *genome presents considerable divergence from those of *Streptomycetes *in gene organization and function. At the same time, the genome sequence confirmed the remarkable potential of *S. erythraea *for producing many other secondary metabolites in addition to erythromycin. Apart for many studies on erythromycin biosynthesis [16,17] and host-vector systems developed for *S. erythraea *[18], there is limited information on the physiology of this strain [19]. In this paper we present the gene expression profiling of 6494 *S. erythraea *ORFs by using a DNA microarray derived from the complete genome sequence of this microorganism. By using a variety of bioinformatic tools we obtained a detailed overview of how *S. erythraea *genome is transcriptionally modulated. These results complement genomic data in deepening our understanding of this industrially relevant strain.

## Results

### Global gene expression during growth of *S. erythraea *NRRL2338

**Figure 1:**
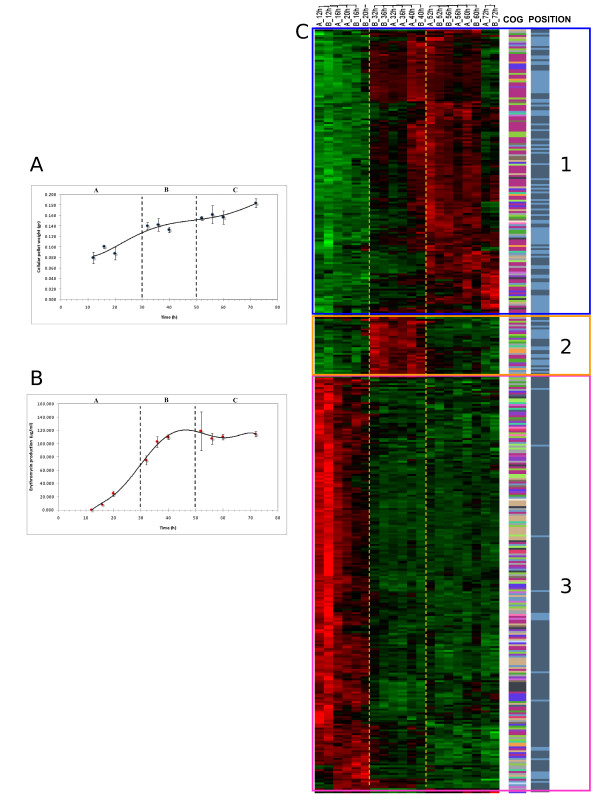
**Global gene expression profiling during the growth time course**. **Panel A) **Graphic representation of how the cellular pellet weight increases along the time course in a sigmoidal way. **Panel B) **Graphic representation of how the erythromycin concentration increases along the time course. **Panel C) **Visualization by dChip of the 404 genes, selected by a q-value <= 0.001, and determining a hierarchical clustering of the samples (Red = up-regulation; Green = down-regulation); on the right side of the figure, two columns respectively show the functional classification of the 404 genes whose transcription profile is visualized and their positional distribution in the core (dark blue) and non core region (light blue) of the genome. The transition from phases A, B and C are evidenced by dotted lines (Phase A: rapid growth lasting until 32 h; Phase B: brief period of growth slowdown lasting until 52 h; Phase C: after 4 hours cultures resume another rapid growth phase from 56 to 72 h before entering the stationary phase).

A time course of *S. erythraea *strain NRRL2338 in SCM medium was monitored following erythromycin production and wet cell weight. Despite the fact that industrial fermentation routinely uses oil, we chose this medium because it does not adversely affect RNA extraction. These results are reported in Figure 1. Erythromycin production was detected after 16 h and increased linearly with time up to 36 h, when it reached a plateau around 100 μg/ml. Cell growth also occurred at a higher rate up to 36–40 h and slowed down after. Stages of growth were defined by change in the rate of increase in cell density (Fig. 1A). An initial period of rapid growth lasting until 32 h (Phase A) was followed by a brief period of growth slowdown lasting until 52 h (Phase B). After 4 hours cultures briefly resumed another rapid growth phase from 56 to 72 h (Phase C) before entering the stationary phase and going towards cellular lysis after about 5 days. RNA samples were extracted at different time points from two independent cultures, processed and hybridized to custom made GeneChips containing DNA oligonucleotide probes corresponding to 6494 predicted *S. erythaea *ORFs.

Gene expression data were first analyzed to identify transcripts modulated during the growth curve. Considering each time point replicate as an independent entry and setting the confidence threshold at q-value ≤ 0.001 (see Methods), the EDGE algorithm identified 404 genes whose expression was statistically modulated during the time course. These transcripts highlighted three distinct large expression clusters, designated as Clusters 1, 2 and 3, and three distinct growth stages, corresponding to the Phases A, B and C described before (Fig. 1C). Cluster 1 consists of 152 genes that are up-regulated during Phase C; Cluster 2 consists of 32 genes whose expression increases during Phase B; and Cluster 3 is made of 220 genes up-regulated during Phase A. Thus, an almost equal number of genes is associated with Clusters 1 and 2 versus Cluster 3. In addition, Clusters 1 and 2 are more related to each other than each of them is to Cluster 3.

The right side of Figure 1C reports, as two separate columns, the functional classification of the 404 genes and their localization in the core and non-core regions of the chromosome. Almost all genes from Cluster 3 belong to the core region, which encodes the majority of functions predicted to be essential, while Clusters 1 and 2 are enriched in genes belonging to the non-core region. No single functional category dominates any Cluster.

Table 1 reports an analysis of the functional categories covered by the 6494 probe-sets and their enrichment or depletion, if any, in the 404 genes with q-value <= 0.001. As expected, categories strongly associated with cellular growth (I.6, Posttranslational modification; II.12, Translation, ribosomal structure; III.5, Energy production and conversion; and III.8, Nucleotide transport and metabolism) were significantly enriched compared to the whole genome. In contrast, categories I.7 (Signal transduction mechanism), II.7 (Replication, recombination and repair) and II.11 (Transcription) were significantly depleted. As can be observed in Table 1, over one third of the genes belong to the categories IV.1 and IV.2 (Function unknown and General function prediction, respectively). Among these 143 genes, less than half (63) gave significant similarity (i.e. e-value < 1E-10) with protein or domains from *S. coelicolor *and/or *Streptomyces avermitilis *[see Additional file 1]. Thus, 80 out of 404 genes whose expression is significantly associated with the time course represent genes of unassigned function unique to *S. erythraea *and absent, or significantly different, in the two *Streptomyces *genomes.

**Table 1:** Functional categories significatively enriched or depleted in the top 404 differentially expressed genes

**COG**	**Functional categories**	**Total probesets**	**Probesets with q-value <= 0.001**
	I. Cellular processes and signalling		
I.1	Cell cycle control, cell division, chromosome partitioning	25	4
I.2	Cell motility	1	0
I.3	Cell wall/membrane/envelope biogenesis	145	13
I.4	Defense mechanisms	63	0
I.5	Intracellular trafficking, secretion, and vesicular transport	17	1
**I.6**	**Posttranslational modification, protein turnover, chaperones**	**134**	**24**
***I.7***	***Signal transduction mechanisms***	***135***	***4***
	II. Information storage and processing		
II.1	Amino acid transport and metabolism	1	0
II.2	Cell wall/membrane/envelope biogenesis	1	0
II.3	Chromatin structure and dynamics	1	0
II.4	General function prediction only	21	2
II.5	Nucleotide transport and metabolism	5	0
II.6	Posttranslational modification, protein turnover, chaperone	3	0
***II.7***	***Replication, recombination and repair***	***172***	***3***
II.8	RNA processing and modification	1	0
II.9	Secondary metabolites biosynthesis, transport catabolism	3	0
II.10	Signal transduction mechanisms	50	2
***II.11***	***Transcription***	***508***	***9***
**II.12**	**Translation, ribosomal structure and biogenesis**	**175**	**17**
	III. Metabolism		
III.1	Amino acid transport and metabolism	466	0
III.2	Carbohydrate transport and metabolism	429	31
III.3	Cell wall/membrane/envelope biogenesis	27	0
III.4	Coenzyme transport and metabolism	186	21
**III.5**	**Energy production and conversion**	**338**	**40**
III.6	Inorganic ion transport and metabolism	189	17
III.7	Lipid transport and metabolism	299	11
**III.8**	**Nucleotide transport and metabolism**	**90**	**12**
III.9	Posttranslational modification, protein turnover, chaperones	6	0
III.10	Secondary metabolites biosynthesis, transport catabolism	205	10
III.11	Signal transduction mechanisms	11	0
	IV. Poorly characterized		
IV.1	Function unknown	2267	110
IV.2	General function prediction only	520	33

The total of the ORFs targeted by the 6494 probe-sets analysed, and the 404 genes with q-value <= 0.001, have been divided into functional categories and the enrichment or depletion of some of them was evidenced. For each functional category **BOLD **= enrichment = % (Probesets with q-value <= 0.001)>2*%(total probesets) ***ITALIC ***= depletion = % (Probesets with q-value <= 0.001)<2*%(total probesets)

### Regional organization of gene expression

**Figure 2:**
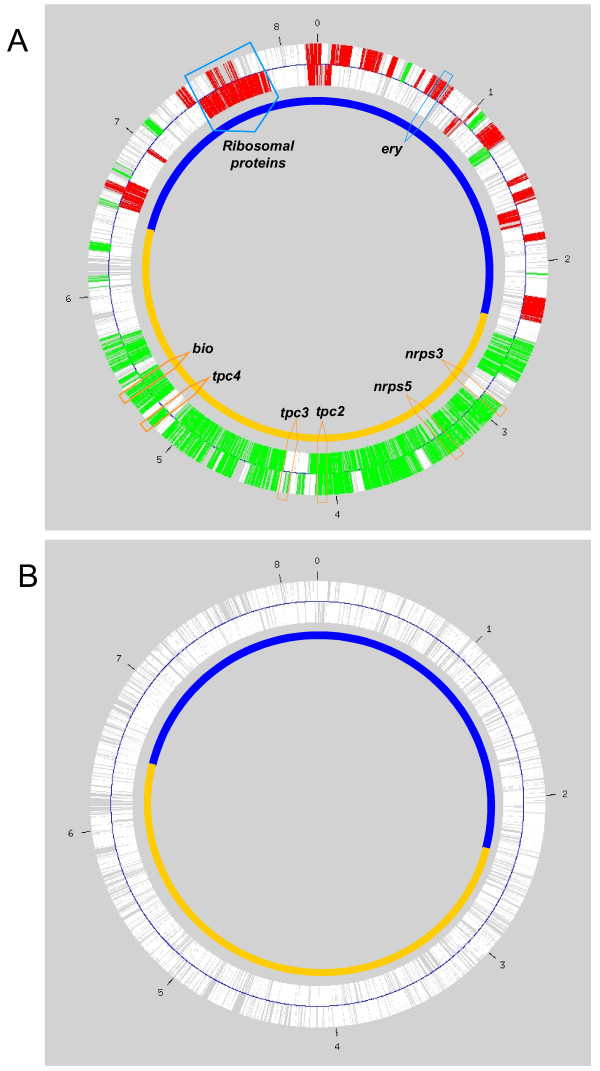
**Regional organization of gene expression**. Visualization of the LAP algorithm results on the two chromosome's strands independently analysed. **Panel A) **Comparison between the transcriptional profiling of all the 6494 *S. erythraea *genes in Phase A versus Phase B. **Panel B) **Comparison between the transcriptional profiling of all the 6494 *S. erythraea *genes in Phase B versus Phase C. A q value of 0.01 and a fold change of 0.5 were chosen as filtering parameters. The transcriptionally up-modulated regions are shown in RED while the down-modulated are in GREEN. The OriC is indicated as O and the resolution of the chromosome is 1 Mb. The core region is evidenced in blue and the non-core region in orange; the clusters involved in the secondary metabolism and discussed in the text are positionally outlined by arrows in figure A.

The *S. coelicolor *genome indicated the existence of a core region, comprising most of the genes deemed to be essential for growth, and a non-core region, highly enriched in the so-called contingency genes [3]. This chromosomal dichotomy has also been reported for *S. avermitilis *[5] and *S. erythraea *[15]. In order to correlate gene expression profiles during growth to genome location of the *S. erythraea *transcripts, we applied the recently developed Locally Adaptive Procedure (LAP), a non-parametric statistical method for identifying chromosomal regions characterized by transcriptional imbalances [20]. LAP uses a locally adaptive smoothing function to integrate gene expression data and the physical position of genes in the genome (see Methods, Data Analysis section). The local adaptation of the smoothing parameters accounts for the heterogeneous distribution of genes along the chromosome, while the application of a statistical approach based on hypothesis testing allows the identification of those chromosomal regions with marked transcriptional variations. Applying the LAP algorithm on the two chromosomal strands independently with 100,000 permutations, we were able to highlight transcriptionally modulated regions from Robust Multi-array Average (RMA) data. Figure 2A illustrates the results of comparing the transcriptional profiles of the 6494 analyzed genes in Phase A versus Phase B, setting *q *value and fold change thresholds to 0.01 and 0.5, respectively. A clear distinction is evident between the core and the non-core regions of the chromosome: the first is characterized by the presence of many over-expressed regions in Phase A and by few islands of down-regulation; while the non-core region shows an opposite trend, being characterized by down-regulated regions during Phase A and by the complete absence of up-regulated islands. It can also be observed from Figure 2A that in the core region either one strand or the other is over-expressed, with the notable exception of the region around *oriC *(position 0 in Fig. 2) and, to a minor extent, the region around 7.3–7.8 Mb, which is characterized mostly by the presence of genes encoding ribosomal proteins. In contrast, both strands show a parallel trend of expression in the non-core region.

A comparison of the transcriptional profiles of Phase B versus Phase C is shown in Figure 2B. In this case, the LAP algorithm did not evidence any region of significant over-expression or down-regulation, confirming the observation that the major changes in regional organization of transcriptional take place in the transition between Phases A and B.

### Expression of paralogous genes

Since *Actinomycetes *genomes contain many sets of paralogs, we wondered whether any genes of predicted similar function were differentially expressed. Selecting only those genes showing at least 70% length identity and at least 60% sequence identity over 70% length, 167 sets of paralogs were identified in the *S. erythraea *genome. Most sets consist of just two genes. Because of the highly stringent criteria for paralogs selection, all proteins in a set are expected to play a substantially similar, if not identical, role in the cell.

**Figure 3:**
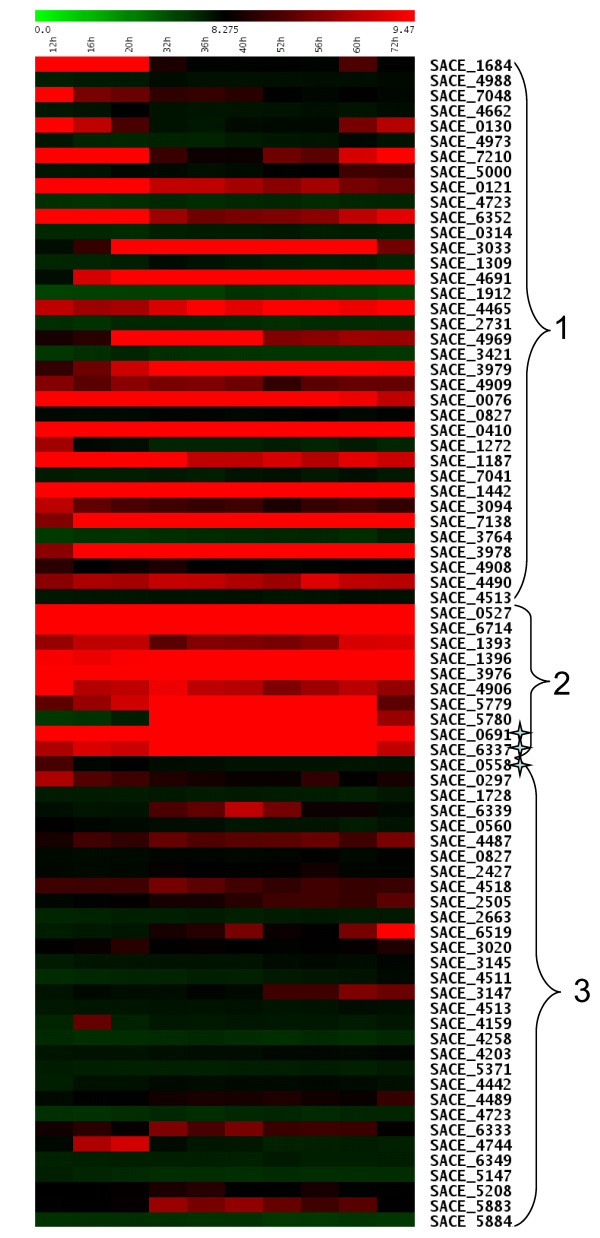
**Expression profiling of paralogous genes along time course**. Expression behaviour of the paralogous couples for which at least one member falls within the 404 clustering genes, resulting in a total of 39 couples. They were visualized by a TMeV heat map in which the paralogs were organized in five main groups. The stars evidence the only set of three paralogs.

The expression patterns were visualized using heat maps created with the TMeV program [21]. Selecting only those sets for which at least one member falls within the 404 top genes led to the identification of 39 sets which could be organized into three main groups (Fig. 3): in Group 1, composed of 18 sets, one gene in each set is up-regulated (either during the entire time course, in Phase A or in Phases B and C), while its paralog is not significantly transcribed. Group 2 consists of 6 sets whose genes show high expression levels during the entire time course. and in Group 3, composed of 15 sets, neither gene in a set is transcribed. Group 2, which may be the most intriguing, includes: SACE_5779-5780, a tandem repeat of carboxypeptidase genes; SACE_3976 and _4906, two CAP family transcription factors associated with the *tcp3 *and *tcp5 *clusters, respectively, of terpene metabolites; SACE_0527 and _6714, encoding two GroEL1 proteins; and SACE_1393 and _1396, and the set represented by SACE_0691, _6337 and _0558, which are all annotated as hypothetical proteins. Thus, among the sets of paralogs analyzed and under the conditions employed, *S. erythraea *usually expresses only one gene from each set of paralogs and does not appear to use different genes within each set under the three growth phases.

### Analysis of functional categories

Having seen that four functional categories are significantly enriched in the 404 differentially expressed genes (Table 1), we analyzed all genes belonging to each of these categories [see Additional file 2, 3]. The I.6 functional category (post-translational modification, protein turnover, chaperones) presents a cluster of 60 genes with increased expression during Phase A and a cluster of 40 genes with the opposite trend. Among this category, it is worth mentioning that SACE_7210, encoding the 70 kDa DnaK chaperonin, is up-regulated during Phase A and, possibly, Phase C, while its paralog SACE_5000 is not expressed. This contrasts with the GroEL chaperonins, both expressed throughout growth (see above).

The genes belonging to the II.12 functional category (translation, ribosomal structure and biogenesis) can be divided into two main clusters. One cluster includes 108 genes up-regulated during Phase A, which mostly encode ribosomal proteins, soluble translation factors and tRNA synthetases. The second cluster is composed of 46 genes having an increased expression in Phases B and C. It is worth mentioning that this cluster includes SACE_5279 and _5431 (encoding ribosomal protein L20 and S1, respectively), SACE_5281 (encoding initiation factor IF3), and SACE_5277 and SACE_5276 (encoding the two subunits of Phe-tRNA synthetase) which, despite the absence of paralogs, are up-regulated during Phases B and C. As observed by Oliynyk et al. [15], *S. erythraea *encodes a full set of protein for the production of selenocysteinyl-tRNA; the specific elongation factor SACE_3551 appears to be expressed during Phases B and C, suggesting that the incorporation of selenocysteine into proteins occurs only after the first rapid growth phase.

The III.5 functional category (energy production and conversion) consists of 88 genes with an increased expression during Phase A and of 110 genes up-regulated in Phase B and C. Particularly relevant is the opposite trend of expression shown by the paralogs SACE_7041/SACE_1187 and SACE_4988/SACE_1684, encoding subunits I and III, respectively, of cytochrome *c *oxidase, and by SACE_2956/SACE_0143 (cytochrome *d *ubiquinol oxidase subunit II), where the first gene in each set is down regulated in Phase A and up-regulated in phases B and C, while the second gene shows the opposite behavior. Other paralogous genes with differential expression in Phase A versus Phases B and C include: SACE_6584/SACE_1170 and SACE_6585/SACE_1171, encoding two subunits of succinate dehydrogenase, SACE_2863/SACE_3267 (coenzyme F420-dependent N(5), N(10)-methylenetetrahydromethanopterin reductase) and SACE_1010/SACE _3563 (4Fe-4S ferredoxin).

The III.4 functional category (coenzyme transport and metabolism), even if not enriched, shows an interesting trend of expression as far as most genes for vitamin and cofactor formation are concerned. While genes for folate, haem and menaquinones biosynthesis are up-regulated during Phase A and later down-regulated, a notable exception is represented by the genes involved in biotin formation, which are up-regulated during Phases B and C. These include the putative operon represented by SACE_4683-4685, as well as SACE_0978 and SACE_5038, which all together are required to convert pimelic acid into biotin. In contrast, no significant changes are observed in the expression of SACE_6501, required for holoenzyme formation through biotinylation of the active site lysine residue of apoenzymes. These results suggest that, under the conditions employed, biotin is probably depleted at the end of Phase A, which requires the cell to synthesize *de novo *this compound, inducing the up-regulation of the *bio *genes in Phases B and C.

### Gene clusters involved in secondary metabolism

**Figure 4:**
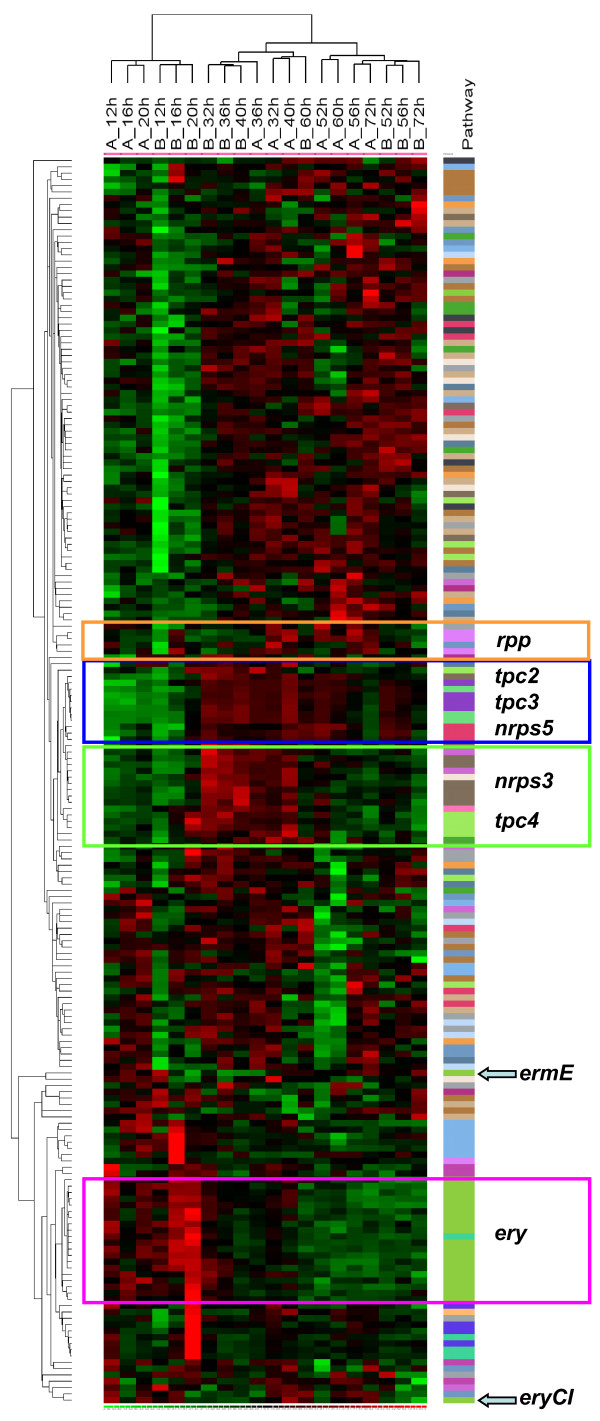
**Gene expression analysis of all the gene clusters involved in secondary metabolism**. dChip visualization of the expression profiling, along the time course, of 196 genes belonging to the clusters for secondary metabolites production (Red = up-regulation; Green = down-regulation). The *ery, tpc2*, *tpc3*, *tpc4*, *nrps3 *and *nrps5 *and *rpp *clusters are evidenced by coloured squared boxes.

The *S. erythraea *genome contains 25 clusters for the biosynthesis of polyketides, terpenes and non-ribosomally synthesized peptides. Table 2 reports the number of specific probesets available for each cluster, including genes for the synthesis of 2,3- dihydroxybenzoyl-AMP. Overall, our probe-sets cover most (196 out of 202) genes involved in secondary metabolism. When the expression profiles of these 196 genes were visualized (Fig. 4), we observed several instances in which genes belonging to the same cluster grouped together. This is particularly evident for the *ery *genes, devoted to erythromycin biosynthesis, as well as for the genes belonging to the clusters designated *tcp2*, *tcp3*, *tcp4*, *nrps3 *and *nrps5*.

**Table 2:** Gene clusters for secondary metabolites production and the number of probe-sets lacking on the Chip

**Clusters**	**Genes number**	**Probeset SACE ID**	**Probeset lacking on Chip**
***Terpenes***			
*tpc1 *(*geo1*)	1	3187	--
*tpc2*	3	3721–3723	--
*tpc3 *(*geo2*)	4	3976–3979	--
*hop*	5	4327–4331	--
*tpc4*	10	4645–4654	--
*tpc5 *(*geo3*)	4	4906–4909	--
***Polyketides***			
*pfa*	11	0018–0028	--
*ery*	21	0712–0721, 0723–0734	--
*rpp*	4	1241–1244	--
*pks1*	6	2342–2347	--
*pks2*	4	2628–2631	--
*pks3*	16	2864–2879	2864
*pke*	18	4128–4145	--
*pks4*	6	4302–4307	--
*pks5*	8	4471–4478	--
*pks6*	12	4567–4578	--
*pks7*	4	5306–5309	--
*pks8*	1	5532	--
***Non-ribosomal peptides***			
*nrps1*	7	1304–1310	1307
*nrps2-pks*	4	2618–1622	2621
*nrps3*	12	2692–2703	--
*nrps4*	5	3013–3017	--
*nrps5*	11	3029–3039	3037–3038
*nrps6*	7	3223–3229	--
*nrps7*	18	4275–4292	4291
			
**Total**	**202**	**Targeted**	**196**

Within the *ery *cluster, 19 genes show a common trend with significant up-regulation during Phase A, consistent with the appearance of antibiotic activity during the early phases of growth (Fig. 1), and a later decrease. The only exceptions to this synchronized behavior are represented by *ermE *gene, the ribosomal methylase involved in erythromycin resistance, and by *eryCI*, encoding the aminotransferase required for desosamine formation. The expression of *eryK*, lying at the opposite end of the *ery *cluster, is instead well-coordinated with the other *ery *genes. It should be noted that expression of SACE_0712, located next to *eryK *and encoding a putative erythromycin esterase, probably inactive because of an IS element insertion [22], is also not coordinated with the expression of the other *ery *genes.

Figure 4 clearly indicates that the clusters *nrps3 *and *tpc4 *are up-regulated during Phase B. The domain composition and the predictive algorithms for substrate specificity of the adenylation domains suggest that *nrps3 *may specify the synthesis of a pentameric siderophore, possibly starting with a 2,3-dihydroxybenzoate unit, for which SACE_2692 through SACE_2696 would be required. The other three co-expressed genes from the *nrps3 *cluster are expected to encode a putative ABC-type Fe^3+^-siderophores transport system (SACE_2697), a putative regulatory protein (SACE_2698) and a hydrolase of the alpha/beta fold family (SACE_2699). It is worth mentioning that SACE_3853 through SACE_3855, directing the synthesis of 2,3-dihydroxybenzoyl-AMP, are also up-regulated in Phase B and totally down-regulated in Phases A and C. These genes may provide the starter unit for the Non Ribosomal Peptide Synthetases (NRPS) specified by *nrps3*. In contrast, SACE_2700 through SACE_2703, which encode hypothetical proteins and transporters, are not coordinately expressed and might not be required for siderophore formation.

The *tcp4 *cluster consists of ten genes, including three putative transcriptional regulators, and specifies the synthesis of an unknown terpenoid which is likely to be modified by multiple hydroxylations (SACE_4643, _4649 and _4651), glycosylation (SACE_4644), amination (SACE_4645) and *O*-methylation (SACE_4650).

The genes from the *nrps5*, *tcp2 *and *tcp3 *clusters appear to be up-regulated during Phases B and C. From the *nrps5 *cluster, the expression profiles indicate that the genes SACE_3033, _3034, _3036 and, possibly, _3030, _3031 and _3035, are up during these phases. This cluster may govern the synthesis of a tetrapeptide siderophore. Of the putative clusters devoted to geosmin formation (*tpc1*, *tpc3 *and *tpc5*), the expression profiles suggest that, at least under our experimental condition, geosmin synthesis is directed by the *tpc3 *cluster, since all four genes assigned to this cluster are up-regulated during these phases. Interestingly, the expression of the *tpc2 *cluster appears also to be coordinated to that of *tpc3*. In this respect, it is worth noticing that the expression of *tpc2 *and *tpc3 *appears to be controlled by SACE_3723 and _3976, each encoding a cyclic nucleotide-binding domain protein. The putative product of the *tpc2 *cluster is currently unknown. Thus, it is likely that the three terpene clusters are each regulated by a representative of the same family of transcriptional regulators, which are apparently activated during Phases B and C.

Finally, the *rpp *cluster, directing the synthesis of the brown pigment [23] appears to be up-regulated during Phases B and C. Consistent with previous results [24], we did not observe any relevant expression of any gene defining the *pke *cluster (SACE_4128 through _4145).

## Discussion

This study represents the first example of analysis of the entire *S. erythraea *transcriptome. The information derived from genome sequencing allowed the design of a microarray to investigate almost all *S. erythraea *ORFs. Thus, we experimentally analyzed gene expression along the time course, determining transcription regional organization, behavior of major functional classes and temporal expression of gene clusters for secondary metabolism. As expected, the use of a DNA microarray specifically designed on the *S. erythraea *genome improved specificity and sensitivity of gene expression analysis, giving a global and at the same time detailed picture of how *S. erythraea *genes are regulated. In their analysis of the differences between wild type and improved production strains, Lum et al. [25] discuss, as one of the major limits of using heterologous microarrays, the inability to monitor expression of genes not present or significantly different in the reference genome. These authors used DNA microarray containing 97% of the *S. coelicolor *ORFs, the erythromycin and tylosin biosynthetic clusters, as well as 40 publicly available *S. erythraea *gene sequences. The data from Oliynik et al. [15] and our own analysis illustrates the limits of performing expression studies using inter-generic microarrays. For example, about 40% of the genes whose expression strongly correlated with the time course, represent sequences unique to *S. erythraea*. Thus, using this microarray we were able to analyze the expression levels of 6494 *S. erythraea *ORFs and to find out which genes determine the transitions between the major three phases which characterize the growth curve under our experimental condition.

Each of the three growth stages, previously observed also with heterologous microarrays [25] and in *S. coelicolor *[11,25], is characterized by a specific gene expression pattern. It should be noted that it is easier to characterize growth stages by the expression profiles of the top 404 genes (Fig. 1C) than by the growth curve (Fig. 1A). Thus, global gene expression analysis enables to distinguish changes in the growth phases of a filamentous microorganism better and more specifically than a crude measurement such as cell weight.

The temporal expression of the *ery *cluster observed here is consistent with the results reported by Reeves et al. [26] and by Lum et al. [25]: under three different experimental conditions, most of the *ery *genes are up-regulated during the first growth phase. Consistently, the *ery *cluster is located in the core region of the chromosome. It should be noted that the *ery *cluster presents a relatively complex transcriptional organization, with at least seven transcriptional units observed (Reeves et al., 1999). Despite this complex transcriptional organization and the lack of a cluster-associated regulator, most *ery *genes are coordinately expressed. It remains to be determined whether expression of the *ery *genes will follow a similar trend in industrially developed strains and/or under different growth conditions.

All other secondary metabolism clusters whose expression was detected under later growth phases are located in the non-core region, including the small operon of *bio *genes. Under the conditions employed, biotin is probably depleted at the end of Phase A, which requires the cell to synthesize *de novo *this compound, inducing the up-regulation of the *bio *genes in Phases B and C. Biotin is required to activate the biotin-dependent carboxylases for the synthesis of malonyl- and methylmalonyl-CoA, the precursors of lipids and multiple methyl-branched lipids, respectively. The cell wall is rich in lipids and high levels of *n*-fatty acids are required during the exponential growth phase to support fast cell membrane production. High levels of biotin may be required during Phases B and C too. One possibility is the *de novo *synthesis of fatty acids required for modifications of the cell wall occurring at the beginning of the stationary phase. Consistently, the *S. erythraea *genome contains multiple acyl-CoA carboxylases and at least some of these genes could be deleted without affecting cell growth or erythromycin production [27]. Alternatively, biotin may be required for other carboxylases (e.g. pyruvate carboxylase) active during Phases B and C.

Despite the fact that our expression analysis was performed under one experimental condition only, six secondary metabolism clusters, in addition to *ery*, are clearly regulated during growth. Among them, *nrps3 *and *nrps5*, likely to direct siderophore formation, are clearly up-regulated during Phases B and C. It remains to be determined whether either of these clusters specifies the formation of erythrobactin, a recently described siderophore of unknown structure [28]. In any case, we were quite surprised to detect their expression in a rich medium such as SCM. Possibly, iron homeostasis is important in *S. erythraea *after the first growth phases has terminated. Currently, the nature of the compound specified by *tpc4 *remains to be determined. Our results clearly point to defined experimental conditions to attempt its characterization. Recently, Gross et al. [29] have described the genome isotopic approach, in which metabolites specified by orphan clusters are identified through a combination of RT-PCR screening and feeding labeled precursors. We would like to suggest that DNA microarrays represent the best way to screen for the expression of orphan clusters. Gene expression in *S. coelicolor *is regionally organized, with genes belonging to the core region up-regulated during the vegetative growth phase [12]. Equivalent results were obtained in this work through the application of the LAP algorithm to the *S. erythraea *gene expression data. The division of the *S. erythraea *chromosome in core and non-core regions, as indicated by our transcriptional profiling, is perfectly consistent with the assignment made on the basis of sequence annotation [15]. The LAP shows not only great sensitivity in determining regional differences in gene expression between core and non-core regions, but also in highlighting strand specific patterns of expression. In fact, two important observations emerge from our analysis. The first is that the non-core region is significantly up-regulated during the transition from Phase A to Phase B, with very small regions of the chromosome being devoid of differential gene expression. The second observation concerns the significant difference between core and non-core regions in terms of strand-specific transcription. Apart from the region around *oriC*, up-regulation occurs mostly on one strand in the core-region, which corresponds to the direction of DNA replication. In contrast, up-regulation during Phases B and C in the non-core region is uniformly distributed and is mostly strand-independent. Thus, our results are consistent with the hypothesis that the non-core region represents a dynamic portion of the chromosome that can readily acquire novel genes which do not undergo the extent of organization in unidirectional transcriptional regions typical of the core region [8].

## Conclusion

This work underlines the importance of using DNA microarrays, specifically designed on the genome sequence of the micro-organism under study, to analyze the coordinate expression of clusters of genes involved in secondary metabolites production even if composed of more transcriptional units. Gene expression profiling by DNA microarray tools coupled with an exhaustive statistical and bioinformatic analysis of the results can be considered the best way to understand the transcriptional organization of the chromosomes of micro-organisms producing natural products, to discover new clusters for secondary metabolites production or, in the case of *S. erythraea *to find out potential antagonist of the cluster for antibiotic production.

## Methods

### Strains, growth conditions and antibiotic production

*S. erythraea *NRRL2338 (ATCC11635) was used as the reference wild type strain. A dense culture of *S. erythraea *grown in oat meal agar was inoculated into SCM medium (20 g soytone, 15 g soluble starch, 10.5 g morpholinepropanesulfonic acid, 1.5 g yeast extract and 0.1 g CaCl_2 _per liter of distilled H_2_O) and grown for 48 h at 33°C and 200 rpm. The culture was then stored as 1-ml frozen aliquots, which were individually used to inoculate three independent 500 ml buffled flasks, each containing 100 ml fresh SCM medium and incubated at 33°C and 200 rpm. From each flask, 1 ml samples were withdrawn at different time points (12, 16, 20, 32, 36, 40, 52, 56, 60 and 72 h from the initial inoculum), separating the mycelium from the broth by centrifugation. The supernatants were used to measure erythromycin production by bioassay against a calibration curve, using *Micrococcus luteus *as test organism.

### Design, construction and validation of the *S. erythraea *microarray

Whole genome shotgun sequence of strain NRRL2338 was obtained using a GS-20 Genome sequencer (454 Life Sciences, Branford, CT) following the manufacturer's standard procedures [30]. Briefly, cells were harvested by centrifugation, DNA was extracted by using the GeneElute™ Bacterial Genomic DNA Kit (SIGMA) and spectrophotometrically quantified. Then, genomic DNA was fragmented by nebulization, ligated to specific adaptors and bound to Sepharose beads to obtain the single strand library, which was diluted and amplified by emulsion PCR. After recovery and washing, DNA beads were loaded onto a PicoTiterPlate where the DNA fragments were sequenced by pyrosequencing. Nine sequencing runs were performed and assembled by means of the Newbler™ Assembler (454 Life Sciences™) yielding 99% of the genome sequence. The DNA sequence was submitted to the TIGR/JCVI Annotation Engine [31], where it was run through TIGR's prokaryotic annotation pipeline. The manual annotation tool Manatee was downloaded from SourceForge [32] and used to manually review the output from the Annotation Engine. The resulting *S. erythraea *genomic sequence was compared with that published by Oliynyk et al. [15] (Accession number: NC_009142) by using the MUMMER bioinformatics tool [33], which indicated that all the contigs obtained perfectly matched against it.

The DNA sequences corresponding to all predicted *S. erythraea *ORFs were submitted to the Affymetrix (Santa Clara, CA) Custom Chip service in order to generate an Expression microarray targeting the whole set of genes. Following Affymetrix internal procedures, a custom chip was generated with 7060 25-mer oligonucleotides probe-sets: 6494 of them were composed of at least 11 probes pairs (Perfect Match/MisMatch); among these, 29 probe-sets, targeting genes longer than 5000 bp, were wider (from 13 to 86 probe pairs); the remaining 566 probe-sets were composed of less than 11 probe pairs. Quality controls of the GeneChip design, construction and validation of the microarrays are integral parts in the Affymetrix GeneChip^® ^array production process (for more detailed information see the following references [34,35]). The analysis was performed on the signals deriving from the 6494 probe-sets containing at least 11 probe pairs. The sequences of all probes present on the GeneChip were then compared with the sequence published by Oliynyk et al. using BLASTN [36]; a perfect match for all of them, both for similarity (100%) and length (25 bp), was found.

### RNA extraction and hybridization

For each time point, RNA was extracted from mycelium pellets deriving from 1-ml culture samples using the GeneElute™ total RNA Purification Kit (SIGMA), recovering it in 50 μl of Elution Solution. After extraction RNAs were quantified with a NanoDrop spectrophotometer (NanoDrop Technologies) and analyzed by capillary electrophoresis on a Agilent Bioanalyzer (Agilent). The RNA samples showing an RIN (RNA Integrity Number, a quality parameter calculated by the instrument software) value higher than 5 were processed for microarray hybridization, following the instructions for "Target Labeling for Prokaryotic GeneChip^® ^Antisense Arrays" (Affymetrix Procaryotic gene Expression Manual). The protocol consists in cDNA synthesis by reverse transcription (starting with 10 μg RNA), followed by cDNA fragmentation with DNase I and labeling with Terminal Deoxynucleotidyl Transferase. The labelled cDNAs were then hybridized for 16 h at 50°C on individual GeneChips. After hybridization, GeneChips were washed and stained with streptavidin-conjugated phycoerythrin by using the Fluidic Station FS450 (Affymetrix) following the ProkGE-WS2v3_450 Protocol. Fluorescent images of the microarrays were acquired using a GeneChip Scanner 3000 (Affymetrix). All Chip images and files have been deposited in the GEO (Gene Expression Omnibus)[37] repository (accession number: GSE9422)

### Data Analysis

The quality of the raw data obtained from microarray hybridization was assessed considering the MAS5.0 (Microarray Suite/Software, Affymetrix) control parameters after a global scaling at a target intensity of 100. Quality and control parameters as well as boxplot of raw intensities indicated the overall high quality of the data set and the absence of any outlying sample. Probe level data was converted to expression values using both the Robust Multi-array Average (RMA) procedure [38] and the MAS5.0 algorithms. In the former case, PM values (Perfect Match) were background-adjusted, normalized using invariant set normalization, and log transformed. In the latter case, intensity levels were normalized using the Global Scaling option to target value (*i.e*. TGT = 100).

Genes characterized by a statistically significant modulation of the expression level during the growth time course (*within-class *temporal differential expression) were identified using the EDGE software package, which is based on the Optimal Discovery Procedure [39] and allows identifying genes that are differentially expressed between two or more different biological conditions or to perform significance analysis on time course experiments [40]. Whereas other methods employ statistics essentially designed for testing one gene at a time (e.g. t-statistics and F-statistics), the ODP uses all relevant information from all genes to test each gene for differential expression, thus improving the power of the test. In the particular case of a time course, ODP takes into account the ordering and spacing information provided by the time points. Briefly, each gene is tested by first fitting a model (e.g. natural cubic sp-lines) under the null hypothesis that there is no differential expression, and then under the alternative hypothesis that there is differential expression. A statistic is calculated to compare the goodness of fit of the two models under the two different hypotheses. The statistic is a quantification of evidence for transcriptional modulation, and the larger it is the more differentially expressed the gene appears to be. Once the statistic is calculated for each gene, a significance cut-off is applied using a false discovery rate criterion [41]. This process, based on the calculation of the null distribution of the statistics when there is no differential expression, is accomplished through a data re-sampling technique and results in the q-value. Modulated genes are finally selected based on the q-value threshold and, eventually on a fold change limit.

Chromosomal regions presenting a *between-class *temporal differential expression were identified using Locally Adaptive Statistical Procedure [20]. LAP is a bioinformatic tool developed under R statistical environment for the identification of differentially expressed chromosomal regions, which accounts for variations in gene distance and density. LAP consists of three main steps: (1) computation of a statistic for ranking probes in order of strength of evidence for an expression feature; (2) adaptive bandwidth smoothing of the statistic after sorting the statistical scores according to the chromosomal position of the corresponding genes; and (3) application of a permutation test to identify differentially expressed chromosomal regions with a q-value correction for multiple tests. Transcriptional and structural information are locally integrated smoothing, along the chromosomal coordinate, an expression statistic. The smoothing procedure is approached as a non-parametric regression problem using a local variable bandwidth kernel estimator. A permutation scheme is used to identify differentially expressed regions under the assumption that each gene has a unique neighborhood and that the corresponding smoothed statistic is not comparable with any statistic smoothed in other regions of the genome. The permutation process over B random assignments allows defining a null smoothed statistic for any chromosomal position. The significance of the differentially expressed regions (i.e. the *p*-value) is computed as the probability that the random null statistic exceeds the observed statistic over B permutations. Once the distribution of empirical *p*-values has been generated, the q-value is used to identify differentially expressed chromosomal regions according to Storey and Tibshirani [41].

Hierarchical clustering and Eisen's maps were used to group modulated genes and samples in the software package dChip [42]. Before clustering, the expression values for a gene across all samples were standardized (linearly scaled) to have mean 0 and standard deviation 1, and these standardized values were used to calculated correlations between genes and samples and served as the basis for merging nodes. Hierarchical agglomerative clustering was carried out using Pearson correlation coefficient as distance metric and centroid as linkage method.

Among the 404 genes with a q-value <= 0.001, those belonging to the functional categories IV.1 and IV.2 (*Function unknown *and *General function prediction only*, respectively) were compared with the *S. coelicolor *or *S. avermitilis *coding sequences using the Pfam database and the hmmpfam bioinformatics tool [43]. To identify the paralog sets present in the *S. erythraea *genome, we tested all the coding sequences against themselves by using the BLASTP program [36] and selected those showing at least 70% length identity and at least 60% sequence identity over 70% length. The expression patterns of the selected paralogs were visualized by using heat maps created with the TMeV program [21].

## Competing interests

The author(s) declare that they have no competing interests.

## Authors' contributions

CP performed the microarray experiments, participated in data analysis and drafted the manuscript. BS performed the data analysis, participated in the manuscript preparation. GC performed the sequence alignment, participated in data analysis and to manuscript preparation. FF performed the data analysis, participated in the manuscript preparation. ER performed the sequencing and participated in sequence alignment. RJPB performed the sequence alignment. BR performed the sequencing and participated in sequence alignment. AA conceived the study and participated in its design and coordination. LRB conceived the study and participated in its design and coordination. SD performed data analysis and drafted the manuscript. GDB conceived the study and participated in its design and coordination.

All authors read and approved the final manuscript.

## Supplementary Material

Additional file 1 List of the genes belonging to the categories IV.1 and IV.2 (Function unknown and General function prediction, respectively) among the top 404, which gave significant similarity with protein or domains from *S. coelicolor *and/or *S. avermitilis* Click here for file

Additional file 2 **Analysis of functional categories**. dChip visualization of the expression pattern along the growth time course of all the genes belonging to the I.6 and III.5 functional categories. The clusters of genes with a transcription trend significantly increased during phase A (from 12 h to 20 h) and decreased in phase B(from 32 h to 52 h) and in phase C (from 56 h to 72 h) are evidenced by coloured squared boxes. Click here for file

Additional file 3 **Analysis of functional categories**. dChip visualization of the expression pattern along the growth time course of all the genes belonging to the II.12, and III.8 functional categories. The clusters of genes with a transcription trend significantly increased during phase A (from 12 h to 20 h) and decreased in phase B(from 32 h to 52 h) and in phase C (from 56 h to 72 h) are evidenced by coloured squared boxes. Click here for file

## References

[B1] Berdy J (2005). Bioactive microbial metabolites. J Antibiot (Tokyo).

[B2] Embley TM, Stackebrandt E (1994). The molecular phylogeny and systematics of the actinomycetes. Annu Rev Microbiol.

[B3] Bentley SD, Chater KF, Cerdeno-Tarraga AM, Challis GL, Thomson NR, James KD, Harris DE, Quail MA, Kieser H, Harper D, Bateman A, Brown S, Chandra G, Chen CW, Collins M, Cronin A, Fraser A, Goble A, Hidalgo J, Hornsby T, Howarth S, Huang CH, Kieser T, Larke L, Murphy L, Oliver K, O'Neil S, Rabbinowitsch E, Rajandream MA, Rutherford K, Rutter S, Seeger K, Saunders D, Sharp S, Squares R, Squares S, Taylor K, Warren T, Wietzorrek A, Woodward J, Barrell BG, Parkhill J, Hopwood DA (2002). Complete genome sequence of the model actinomycete Streptomyces coelicolor A3(2). Nature.

[B4] Omura S, Ikeda H, Ishikawa J, Hanamoto A, Takahashi C, Shinose M, Takahashi Y, Horikawa H, Nakazawa H, Osonoe T, Kikuchi H, Shiba T, Sakaki Y, Hattori M (2001). Genome sequence of an industrial microorganism Streptomyces avermitilis: deducing the ability of producing secondary metabolites. Proc Natl Acad Sci U S A.

[B5] Ikeda H, Ishikawa J, Hanamoto A, Shinose M, Kikuchi H, Shiba T, Sakaki Y, Hattori M, Omura S (2003). Complete genome sequence and comparative analysis of the industrial microorganism Streptomyces avermitilis. Nat Biotechnol.

[B6] Donadio S, Monciardini P, Sosio M (2007). Polyketide synthases and nonribosomal peptide synthetases: the emerging view from bacterial genomes. Nat Prod Rep.

[B7] Udwary DW, Zeigler L, Asolkar RN, Singan V, Lapidus A, Fenical W, Jensen PR, Moore BS (2007). Genome sequencing reveals complex secondary metabolome in the marine actinomycete Salinispora tropica. Proc Natl Acad Sci U S A.

[B8] Challis GL, Hopwood DA (2003). Synergy and contingency as driving forces for the evolution of multiple secondary metabolite production by Streptomyces species. Proc Natl Acad Sci U S A.

[B9] Hopwood DA (1999). Forty years of genetics with Streptomyces: from in vivo through in vitro to in silico. Microbiology.

[B10] DeRisi JL, Iyer VR, Brown PO (1997). Exploring the metabolic and genetic control of gene expression on a genomic scale. Science.

[B11] Huang J, Lih CJ, Pan KH, Cohen SN (2001). Global analysis of growth phase responsive gene expression and regulation of antibiotic biosynthetic pathways in Streptomyces coelicolor using DNA microarrays. Genes Dev.

[B12] Karoonuthaisiri N, Weaver D, Huang J, Cohen SN, Kao CM (2005). Regional organization of gene expression in Streptomyces coelicolor. Gene.

[B13] Chater KF (1990). The improving prospects for yield increase by genetic engineering in antibiotic-producing Streptomycetes. Biotechnology (N Y).

[B14] Sezonov G, Blanc V, Bamas-Jacques N, Friedmann A, Pernodet JL, Guerineau M (1997). Complete conversion of antibiotic precursor to pristinamycin IIA by overexpression of Streptomyces pristinaespiralis biosynthetic genes. Nat Biotechnol.

[B15] Oliynyk M, Samborskyy M, Lester JB, Mironenko T, Scott N, Dickens S, Haydock SF, Leadlay PF (2007). Complete genome sequence of the erythromycin-producing bacterium Saccharopolyspora erythraea NRRL23338. Nat Biotechnol.

[B16] Katz L, Donadio S (1995). Macrolides. Biotechnology.

[B17] Staunton J, Weissman KJ (2001). Polyketide biosynthesis: a millennium review. Nat Prod Rep.

[B18] Gaisser S, Reather J, Wirtz G, Kellenberger L, Staunton J, Leadlay PF (2000). A defined system for hybrid macrolide biosynthesis in Saccharopolyspora erythraea. Mol Microbiol.

[B19] Vanden Boom TJ (2000). Recent developments in the molecular genetics of the erythromycin-producing organism Saccharopolyspora erythraea. Adv Appl Microbiol.

[B20] Callegaro A, Basso D, Bicciato S (2006). A locally adaptive statistical procedure (LAP) to identify differentially expressed chromosomal regions. Bioinformatics.

[B21] Saeed AI, Sharov V, White J, Li J, Liang W, Bhagabati N, Braisted J, Klapa M, Currier T, Thiagarajan M, Sturn A, Snuffin M, Rezantsev A, Popov D, Ryltsov A, Kostukovich E, Borisovsky I, Liu Z, Vinsavich A, Trush V, Quackenbush J (2003). TM4: a free, open-source system for microarray data management and analysis. Biotechniques.

[B22] Pereda A, Summers R, Katz L (1997). Nucleotide sequence of the ermE distal flank of the erythromycin biosynthesis cluster in Saccharopolyspora erythraea. Gene.

[B23] Cortes J, Velasco J, Foster G, Blackaby AP, Rudd BA, Wilkinson B (2002). Identification and cloning of a type III polyketide synthase required for diffusible pigment biosynthesis in Saccharopolyspora erythraea. Mol Microbiol.

[B24] Boakes S, Oliynyk M, Cortes J, Bohm I, Rudd BA, Revill WP, Staunton J, Leadlay PF (2004). A new modular polyketide synthase in the erythromycin producer Saccharopolyspora erythraea. J Mol Microbiol Biotechnol.

[B25] Lum AM, Huang J, Hutchinson CR, Kao CM (2004). Reverse engineering of industrial pharmaceutical-producing actinomycete strains using DNA microarrays. Metab Eng.

[B26] Reeves AR, English RS, Lampel JS, Post DA, Vanden Boom TJ (1999). Transcriptional organization of the erythromycin biosynthetic gene cluster of Saccharopolyspora erythraea. J Bacteriol.

[B27] Donadio S, Staver MJ, Katz L (1996). Erythromycin production in Saccharopolyspora erythraea does not require a functional propionyl-CoA carboxylase. Mol Microbiol.

[B28] Oliveira PH, Batagov A, Ward J, Baganz F, Krabben P (2006). Identification of erythrobactin, a hydroxamate-type siderophore produced by Saccharopolyspora erythraea. Lett Appl Microbiol.

[B29] Gross H, Stockwell VO, Henkels MD, Nowak-Thompson B, Loper JE, Gerwick WH (2007). The genomisotopic approach: a systematic method to isolate products of orphan biosynthetic gene clusters. Chem Biol.

[B30] Margulies M, Egholm M, Altman WE, Attiya S, Bader JS, Bemben LA, Berka J, Braverman MS, Chen YJ, Chen Z, Dewell SB, Du L, Fierro JM, Gomes XV, Godwin BC, He W, Helgesen S, Ho CH, Irzyk GP, Jando SC, Alenquer ML, Jarvie TP, Jirage KB, Kim JB, Knight JR, Lanza JR, Leamon JH, Lefkowitz SM, Lei M, Li J, Lohman KL, Lu H, Makhijani VB, McDade KE, McKenna MP, Myers EW, Nickerson E, Nobile JR, Plant R, Puc BP, Ronan MT, Roth GT, Sarkis GJ, Simons JF, Simpson JW, Srinivasan M, Tartaro KR, Tomasz A, Vogt KA, Volkmer GA, Wang SH, Wang Y, Weiner MP, Yu P, Begley RF, Rothberg JM (2005). Genome sequencing in microfabricated high-density picolitre reactors. Nature.

[B31] TIGR/JCVI Annotation Engine. http://www.tigr.org/AnnotationEngine.

[B32] Manatee. http://manatee.sourceforge.net.

[B33] MUMMER. http://mummer.sourceforge.net.

[B34] Affymetrix Manufacturing Quality Technote. http://www.affymetrix.com/support/technical/technotes/manufacturing_quality_technote.pdf.

[B35] Affymetrix Custom GeneChip program design. http://www.affymetrix.com/support/technical/datasheets/customseq_program_datasheet.pdf.

[B36] Altschul SF, Gish W, Miller W, Myers EW, Lipman DJ (1990). Basic local alignment search tool. J Mol Biol.

[B37] GEO (Gene Expression Omnibus). http://www.ncbi.nlm.nih.gov/geo/info/linking.html..

[B38] Irizarry RA, Hobbs B, Collin F, Beazer-Barclay YD, Antonellis KJ, Scherf U, Speed TP (2003). Exploration, normalization, and summaries of high density oligonucleotide array probe level data. Biostatistics.

[B39] Storey JD, Dai JY, Leek JT (2007). The optimal discovery procedure for large-scale significance testing, with applications to comparative microarray experiments. Biostatistics.

[B40] Storey JD, Xiao W, Leek JT, Tompkins RG, Davis RW (2005). Significance analysis of time course microarray experiments. Proc Natl Acad Sci U S A.

[B41] Storey JD, Tibshirani R (2003). Statistical significance for genomewide studies. Proc Natl Acad Sci U S A.

[B42] Li C, Wong WH (2001). Model-based analysis of oligonucleotide arrays: expression index computation and outlier detection. Proc Natl Acad Sci U S A.

[B43] Pfam. http://pfam.sanger.ac.uk/.

