# Supplementary material for: Complete gene expression profiling of Saccharopolyspora erythraea using GeneChip DNA microarrays
Source: Microb Cell Fact. 2007 Nov 26;6:37. doi: 10.1186/1475-2859-6-37 (PMC2206050; doi:10.1186/1475-2859-6-37)
Supplement: Additional file 1 — List of the genes belonging to the categories IV.1 and IV.2 (Function unknown and General function prediction, respectively) among the top 404, which gave significant similarity with protein or domains from S. coelicolor and/or S. avermitilis [file 1475-2859-6-37-S1.doc]

**Additional file 2**

| **Probeset ID** | **Gene Symbol** | **Product** | **Domain** | **Organism** | **e-value** |
| --- | --- | --- | --- | --- | --- |
| SACE_6321 | - | probable ATP-binding component of ABC transporter | ABC_tran | *S. coelicolor, S. avermitilis* | 2E-98 |
| SACE_6339 | cvnD6 | ATP/GTP-binding protein | ATP_bind_1 | *S. coelicolor, S. avermitilis* | 3E-64 |
| SACE_3239 | slpD | proteinase (secreted protein) | Abhydrolase_1 | *S. coelicolor, S. avermitilis* | 2E-13 |
| SACE_5892 | - | secreted peptidase | Abhydrolase_1 | *S. coelicolor, S. avermitilis* | 6E-11 |
| SACE_6763 | - | alpha/beta hydrolase fold | Abhydrolase_1 | *S. coelicolor, S. avermitilis* | 7E-20 |
| SACE_3239 | slpD | proteinase (secreted protein) | Abhydrolase_4 | *S. coelicolor, S. avermitilis* | 1E-25 |
| SACE_5892 | - | secreted peptidase | Abhydrolase_4 | *S. coelicolor, S. avermitilis,* | 8E-27 |
| SACE_5966 | - | GCN5-related N-acetyltransferase | Acetyltransf_1 | *S. coelicolor, S. avermitilis,* | 3E-12 |
| SACE_7048 | - | 2,5-diketo-D-gluconic acid reductase | Aldo_ket_red | *S. coelicolor, S. avermitilis,* | 3E-133 |
| SACE_1647 | - | hypothetical protein | BNR | *S. coelicolor, S. avermitilis* | 3E-14 |
| SACE_2505 | - | peptidase M50 | CBS | *S. coelicolor, S. avermitilis* | 1E-29 |
| SACE_3157 | - | putative signal-transduction protein with CBS domains | CBS | *S. coelicolor, S. avermitilis* | 3E-29 |
| SACE_3488 | - | CBS domain protein | CBS | *S. coelicolor, S. avermitilis* | 4E-29 |
| SACE_1201 | - | ATP-dependent Clp protease adaptor protein ClpS | ClpS | *S. coelicolor, S. avermitilis* | 3E-30 |
| SACE_0073 | - | probable copper resistance protein | CopC | *S. coelicolor, S. avermitilis* | 5E-16 |
| SACE_2391 | - | creatininase | Creatininase | *S. coelicolor, S. avermitilis* | 2E-49 |
| SACE_3260 | - | hypothetical protein | Cupin_5 | *S. coelicolor, S. avermitilis* | 2E-11 |
| SACE_1432 | - | hypothetical protein | DUF143 | *S. coelicolor, S. avermitilis* | 2E-48 |
| SACE_0335 | - | protein of unknown function DUF1469 | DUF1469 | *S. coelicolor, S. avermitilis* | 1E-35 |
| SACE_3825 | - | sulfatase modifying factor | DUF323 | *S. coelicolor, S. avermitilis* | 3E-94 |
| SACE_1750 | - | hypothetical protein | DUF349 | *S. coelicolor, S. avermitilis* | 4E-51 |
| SACE_5956 | - | hypothetical protein | DUF552 | *S. coelicolor, S. avermitilis* | 7E-16 |
| SACE_2002 | - | protein of unknown function DUF574 | DUF574 | *S. coelicolor, S. avermitilis* | 1E-109 |
| SACE_5365 | - | hypothetical protein | DUF574 | *S. coelicolor, S. avermitilis* | 5E-87 |
| SACE_6394 | - | protein of unknown function DUF59 | DUF59 | *S. coelicolor, S. avermitilis* | 2E-13 |
| SACE_6338 | - | hypothetical protein | DUF742 | *S. coelicolor, S. avermitilis* | 7E-33 |
| SACE_1291 | - | ErfK/YbiS/YcfS/YnhG | ErfK_YbiS_YhnG | *S. coelicolor, S. avermitilis* | 1E-35 |
| SACE_5414 | - | hypothetical protein | Erythro_esteras | *S. coelicolor, S. avermitilis* | 6E-10 |
| SACE_5226 | - | hypothetical protein | Glyco_hydro_43 | *S. coelicolor, S. avermitilis* | 4E-12 |
| SACE_2077 | - | putative transcriptional regulator (DNA-binding protein) | HTH_3 | *S. coelicolor, S. avermitilis,* | 3E-11 |
| SACE_0920 | - | channel protein, hemolysin III family | HlyIII | *S. coelicolor, S. avermitilis* | 2E-59 |
| SACE_4489 | - | probable hydroxyacylglutathione hydrolase | Lactamase_B | *S. coelicolor, S. avermitilis* | 3E-31 |
| SACE_0311 | - | metallophosphoesterase | Metallophos | *S. coelicolor, S. avermitilis* | 7E-11 |
| SACE_1089 | - | peptidase M16-like | Peptidase_M16_C | *S. coelicolor, S. avermitilis* | 2E-20 |
| SACE_1090 | - | peptidase M16-like | Peptidase_M16_C | *S. coelicolor, S. avermitilis* | 8E-27 |
| SACE_5205 | - | aminopeptidase (secreted protein) | Peptidase_M28 | *S. coelicolor, S. avermitilis,* | 1E-59 |
| SACE_2505 | - | peptidase M50 | Peptidase_M50 | *S. coelicolor, S. avermitilis* | 3E-18 |
| SACE_1697 | - | cyclase/dehydrase | Polyketide_cyc | *S. coelicolor, S. avermitilis,* | 2E-15 |
| SACE_0504 | - | serine protease precursor | Pro_Al_protease | *S. coelicolor, S. avermitilis, ,* | 1E-12 |
| SACE_3145 | fprC | ferredoxin reductase | Pyr_redox | *S. coelicolor, S. avermitilis,* | 2E-23 |
| SACE_4511 | fprC | ferredoxin reductase | Pyr_redox | *S. coelicolor, S. avermitilis,* | 4E-26 |
| SACE_3145 | fprC | ferredoxin reductase | Pyr_redox_2 | *S. coelicolor, S. avermitilis, ,* | 1E-28 |
| SACE_4511 | fprC | ferredoxin reductase | Pyr_redox_2 | *S. coelicolor, S. avermitilis, ,* | 1E-28 |
| SACE_4996 | - | hypothetical protein | Pyridox_oxidase | *S. coelicolor, S. avermitilis* | 1E-20 |
| SACE_0558 | cvnB7 | hypothetical protein | Robl_LC7 | *S. coelicolor, S. avermitilis* | 2E-28 |
| SACE_1954 | cvnB11 | hypothetical protein | Robl_LC7 | *S. coelicolor, S. avermitilis* | 2E-28 |
| SACE_0121 | - | SSS sodium solute transporter superfamily | SSF | *S. coelicolor, S. avermitilis* | 3E-101 |
| SACE_0706 | - | putative S. subtilisin inhibitor-like protein | SSI | *S. coelicolor, S. avermitilis, ,* | 2E-16 |
| SACE_2698 | - | putative regulatory protein | TauD | *S. coelicolor, S. avermitilis,* | 1-E-11 |
| SACE_0298 | - | hypothetical protein | TetR_N | *S. coelicolor, S. avermitilis, ,* | 1E-15 |
| SACE_6239 | - | putative transcriptional regulator | TetR_N | *S. coelicolor, S. avermitilis, ,* | 8E-17 |
| SACE_5275 | - | hypothetical protein | Trypsin | *S. coelicolor, S. avermitilis, ,* | 8E-10 |
| SACE_0312 | - | GatB/YqeY | YqeY | *S. coelicolor, S. avermitilis* | 5E-19 |
| SACE_0691 | cvnB1 | hypothetical protein | Robl_LC7 | *S. coelicolor, S. avermitilis* | 3.1E-21 |
| SACE_3723 | - | cyclic nucleotide-binding domain (cNMP-BD) protein | cNMP_binding | *S. coelicolor, S. avermitilis* | 6E-16 |
| SACE_3977 | - | terpene synthase, metal binding domain protein | Terpene_synth_C | *S. coelicolor, S. avermitilis* | 4E-19 |
| SACE_3978 | - | cyclic nucleotide-binding domain (cNMP-BD) protein | cNMP_binding | *S. coelicolor, S. avermitilis* | 6E-17 |
| SACE_4894 | - | hydrolase, alpha/beta fold family | Abhydrolase_1 | *S. coelicolor, S. avermitilis* | 2E-16 |
| SACE_4969 | - | hypothetical protein | DUF839 | *S. coelicolor, S. avermitilis* | 9E-27 |
| SACE_6065 | - | putative muramoyl-pentapeptide carboxypeptidase | Peptidase_M15_3PG_binding_1 | *S. coelicolor, S. avermitilis* | 2E-26 3E-15 |
| SACE_6395 | - | amidohydrolase 2 | Amidohydro_2 | *S. coelicolor, S. avermitilis* | 1E-35 |
| SACE_6396 | - | alcohol dehydrogenase, zinc-binding | ADH_NADH_zinc_N | *S. coelicolor, S. avermitlis, ,* | 1E-48 2E-31 |
| SACE_6506 | - | serine protease precursor | Trypsin | *S. coelicolor, S. avermitilis, ,* | 9E-17 |
