# Supplementary figures and images for: Complete gene expression profiling of Saccharopolyspora erythraea using GeneChip DNA microarrays
Source: Microb Cell Fact. 2007 Nov 26;6:37. doi: 10.1186/1475-2859-6-37 (PMC2206050; doi:10.1186/1475-2859-6-37)

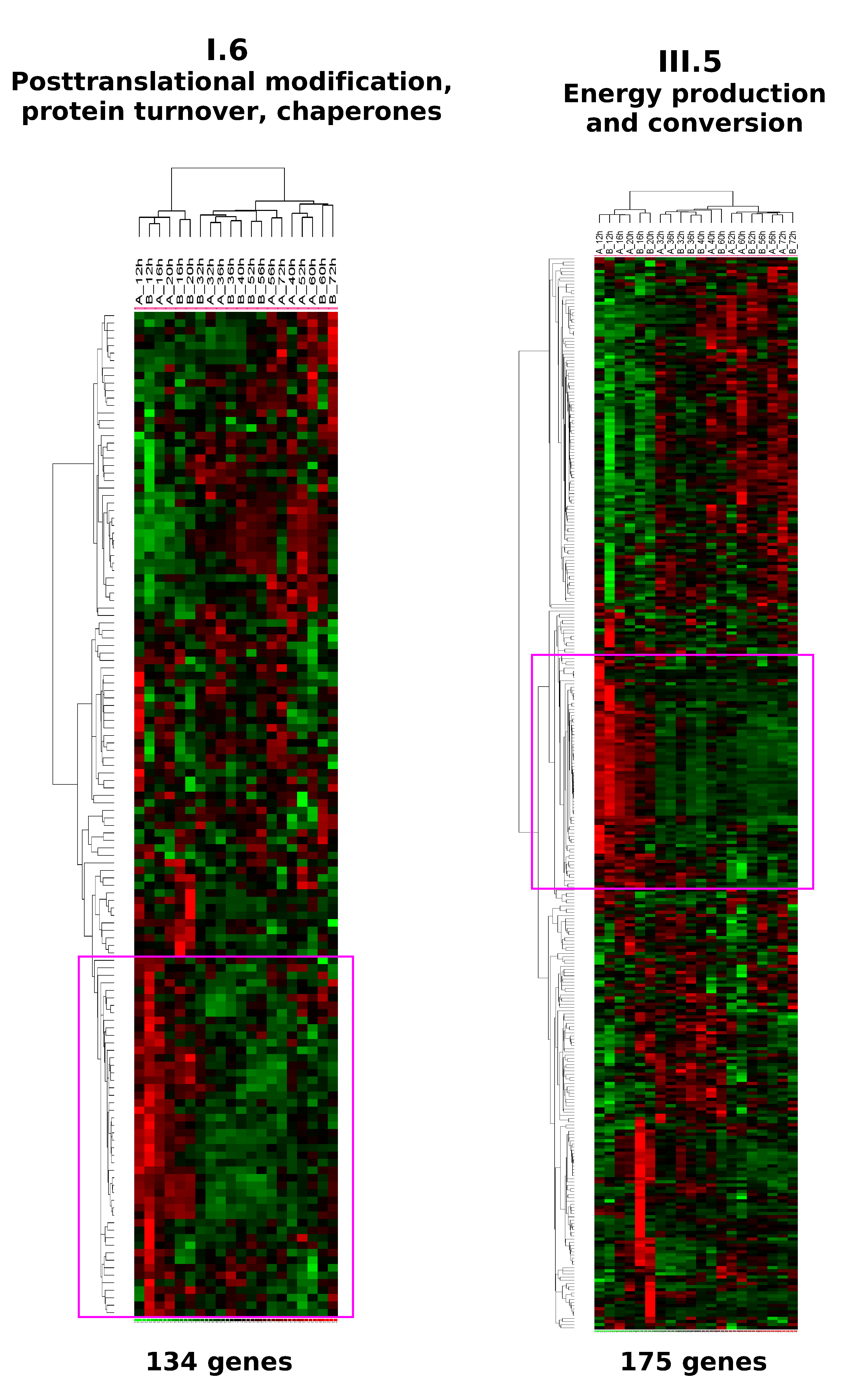

Supplement: Additional file 2 — Analysis of functional categories. dChip visualization of the expression pattern along the growth time course of all the genes belonging to the I.6 and III.5 functional categories. The clusters of genes with a transcription trend significantly increased during phase A (from 12 h to 20 h) and decreased in phase B(from 32 h to 52 h) and in phase C (from 56 h to 72 h) are evidenced by coloured squared boxes. [file 1475-2859-6-37-S2.png]

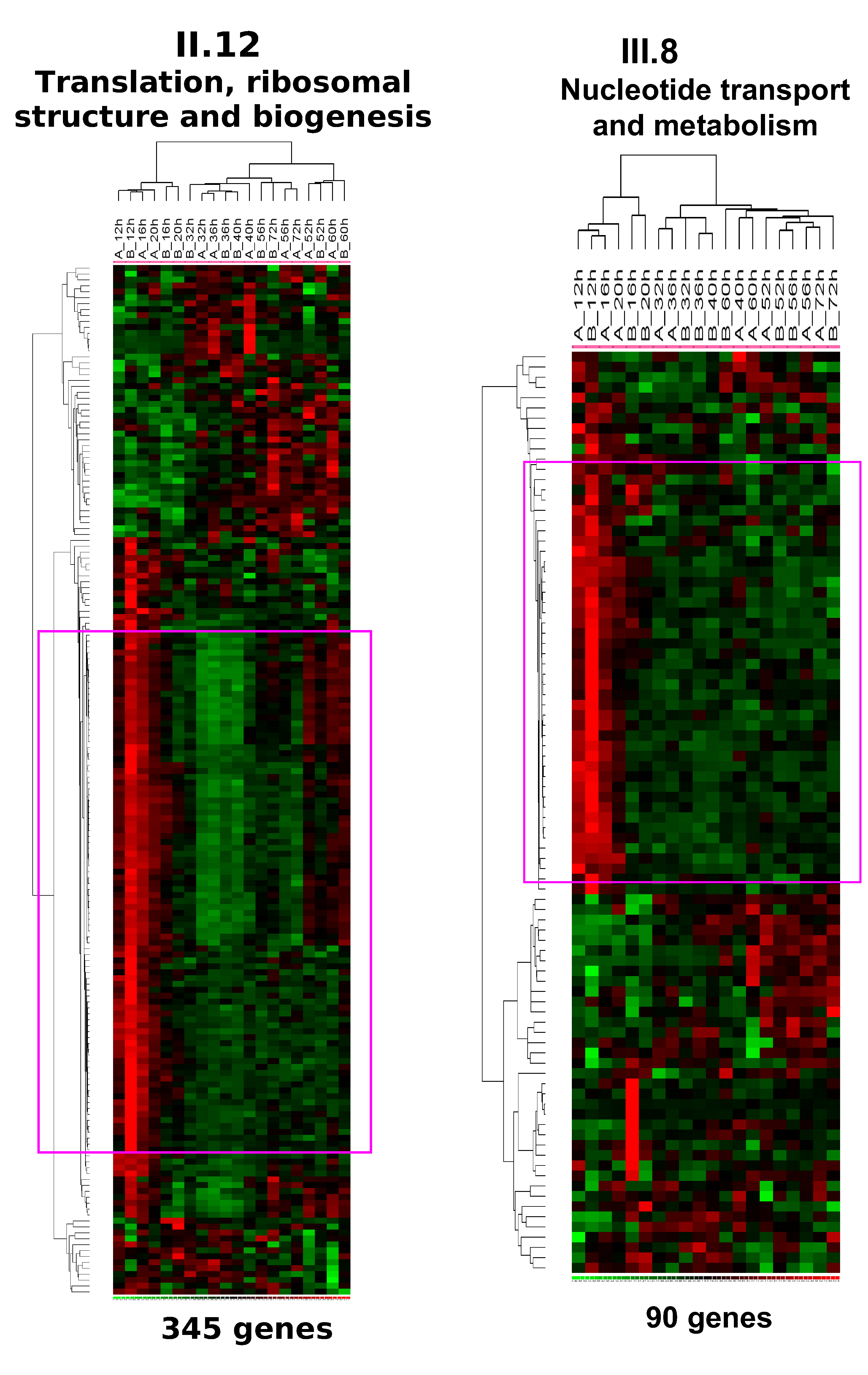

Supplement: Additional file 3 — Analysis of functional categories. dChip visualization of the expression pattern along the growth time course of all the genes belonging to the II.12, and III.8 functional categories. The clusters of genes with a transcription trend significantly increased during phase A (from 12 h to 20 h) and decreased in phase B(from 32 h to 52 h) and in phase C (from 56 h to 72 h) are evidenced by coloured squared boxes. [file 1475-2859-6-37-S3.png]
